# Study of Outcomes of Mucormycosis in COVID-19 Patients at a Tertiary Care Hospital in Central India: A Retrospective Study

**DOI:** 10.7759/cureus.75728

**Published:** 2024-12-15

**Authors:** Vinay Meshram, Madhavi Madkey, Atul Rajkondawar

**Affiliations:** 1 Department of Medicine, Government Medical College and Hospital, Nagpur, IND; 2 Department of Microbiology, All India Institute of Medical Sciences, Raipur, IND

**Keywords:** amphotericin b, corticosteroids, covid-19, diabetes mellitus, mucormycosis, surgical debridement

## Abstract

Background

The COVID-19 pandemic has posed unprecedented challenges to the global healthcare system. Among the various complications, mucormycosis, a fungal infection caused by the Mucorales order, has emerged as a significant threat, particularly in immunocompromised individuals. This study aims to evaluate the outcomes of mucormycosis in COVID-19 patients treated at a tertiary care hospital in Central India.

Method

This retrospective study reviewed the medical records of 72 patients diagnosed with mucormycosis following COVID-19 infection between April 2021 and July 2021 at the tertiary care hospital. Data on demographics, clinical features, comorbidities, treatment received (surgery, antifungal medications), and treatment outcomes (mortality, response to treatment) were collected and analyzed.

Results

The mean age of patients was 55.42±12.31 years, with a male predominance (n=44; 61.11%). Facial pain (n=61; 84.72%) and headache (n=58; 80.55%) were the most common clinical features. The mean duration of symptoms was 14.31±5.4 days. Steroids were used in the majority of the patients (n=50; 69%). The most common comorbidity was diabetes mellitus (n=42; 58.33%). Out of 72 patients, 40 (55.6%) survived and 32 (44.4%) patients died. Surgical debridement was performed in 45 (62.5%) patients. All 72 patients (100%) received amphotericin B, an antifungal medication. The older age and lack of early surgical intervention were significant factors associated with higher mortality in patients with mucormycosis in COVID-19.

Conclusion

This retrospective study reinforces the critical role of early diagnosis, immediate systemic antifungal therapy, stringent management of comorbidities, and prompt surgical intervention in improving outcomes for mucormycosis in COVID-19 patients.

## Introduction

The COVID-19 pandemic, caused by the novel coronavirus SARS-CoV-2, has led to significant morbidity and mortality worldwide [1]. While the primary manifestations of COVID-19 involve the respiratory system, the disease has also been associated with a range of secondary infections, including bacterial, viral, and fungal infections. The immunocompromised state induced by severe COVID-19, coupled with the use of immunosuppressive therapies, has been implicated in the increased incidence of these infections [2]. Among fungal infections, mucormycosis, a rare but serious fungal infection caused by molds in the order Mucorales, has emerged as a notable complication, particularly in immunocompromised individuals [3].

Mucormycosis is a rare fungal infection caused by the fungi of order Mucorales. Mucorales species most frequently recovered from clinical specimens include Rhizopus, Lichtheimia, and Mucor, while species of other Zygomycetes genera, namely Rhizomucor, Saksenaea, Cunninghamella, and Apophysomyces, are relatively less common. The infection primarily affects individuals with underlying health conditions such as diabetes mellitus, malignancies, solid organ transplantation, prolonged neutropenia, and the use of corticosteroids and other immunosuppressive agents. The pathogenesis of mucormycosis involves the inhalation of fungal spores, which then invade the host tissues, leading to severe tissue necrosis and, if untreated, high mortality rates. The infection can present in various forms, including rhino-orbital-cerebral, pulmonary, gastrointestinal, cutaneous, and disseminated mucormycosis, each with distinct clinical manifestations and implications [3,4].

However, the relationship between COVID-19 and mucormycosis is multifaceted [5]. COVID-19 itself induces a state of immune dysregulation characterized by lymphopenia, cytokine storm, and impaired immune responses [6], making patients more susceptible to secondary infections. Furthermore, the widespread use of corticosteroids and immunomodulatory therapies to manage severe COVID-19 has been implicated in the increased incidence of secondary fungal infections, including mucormycosis. Corticosteroids, while reducing inflammation, also impair the immune response and elevate blood glucose levels, creating an environment conducive to fungal proliferation [7,8].

Several studies have reported an alarming rise in cases of COVID-19-associated mucormycosis, particularly in countries like India, where there is a high prevalence of diabetes mellitus and other risk factors [9-11]. The synergistic effect of COVID-19 and mucormycosis has posed significant challenges to healthcare systems, necessitating prompt diagnosis and comprehensive treatment strategies to mitigate the high morbidity and mortality associated with this dual infection. Moreover, the surge in mucormycosis cases during the COVID-19 pandemic has highlighted the urgent need for in-depth studies to understand the clinical characteristics, risk factors, treatment outcomes, and prognostic indicators of this dual infection [11]. This retrospective study aims to evaluate the outcomes of mucormycosis in COVID-19 patients treated at a tertiary care hospital in Central India.

## Materials and methods

This retrospective study reviewed the medical records of 72 patients diagnosed with mucormycosis following COVID-19 infection between April 2021 and July 2021 at the tertiary care hospital. Clinical histories and treatment details of the patients were documented using pre-designed forms. Various specimens such as nasal swabs, endotracheal tube secretion, sputum, and tissues were collected from in-patient departments such as the ICU and COVID-19 ward for the detection of fungal infection in the specimens. Diagnosis of invasive rhino-orbited mucormycosis was made by characteristic clinical features and the potassium hydroxide (KOH) mount showing broad aseptate fungal hyphae. Lactophenol cotton blue mount was prepared to confirm the findings. Other methods used for tissue diagnosis were immunohistochemistry and polymerase chain reaction (PCR). Fungal culture was done on Sabouraud dextrose agar wherever required. CT and/or MRI of the paranasal sinuses, orbits, and brain were done. A detailed retrospective analysis of confirmed mucormycosis cases was done. Data on demographics, clinical features, underlying medical conditions (diabetes, etc.), COVID-19 disease severity, diagnostic methods, treatment received (surgery, antifungal medications), and treatment outcomes (mortality, response to treatment) were collected and analyzed.

The data were collected and entered in a Microsoft Excel (Microsoft Corp., Redmond, US) sheet and then statistically analyzed using SPSS (IBM, Armonk, US) version 20.0. Continuous variables were expressed as mean ± SD and categorical variables were summarized as frequencies and percentages. One-way ANOVA was used to compare continuous variables, and the chi-square test was used for categorical variables. P-value <0.05 was considered statistically significant.

## Results

A total of 72 patients diagnosed with mucormycosis following COVID-19 infection were studied. Most of the patients were from the age group of 51 to 60 years (n=34; 47.22%) with a mean age of 55.42±12.31 years ranging from 21 to 76 years with a male predominance (n=44; 61.11%), as shown in Table 1.

**Table 1:** Demographic data of the patients These categorical variables were summarized as frequencies and percentages.

Age group in years	Male (n)	Female (n)	Total n (%)
21 to 30	1	-	1 (1.38%)
31 to 40	4	2	6 (8.33%)
41 to 50	10	6	16 (22.22%)
51 to 60	21	13	34 (47.22%)
>60	8	7	15 (20.83%)
Total n (%)	44 (61.11%)	28 (38.88%)	72 (100.0%)

**Figure 1:**
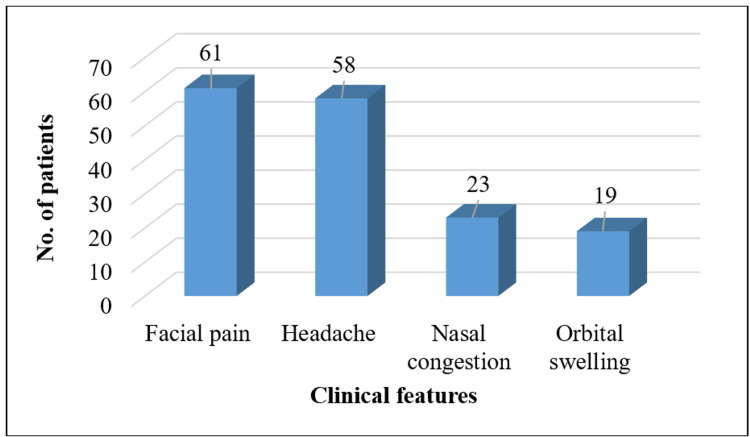
Clinical features of the patients These categorical variables were summarized as frequencies.

Facial pain (n=61; 84.72%) and headache (n=58; 80.55%) were the most common clinical features, as depicted in Figure 1. The mean duration of symptoms was 14.31±5.4 days. Steroids were used in 50 (69%) patients.

**Figure 2:**
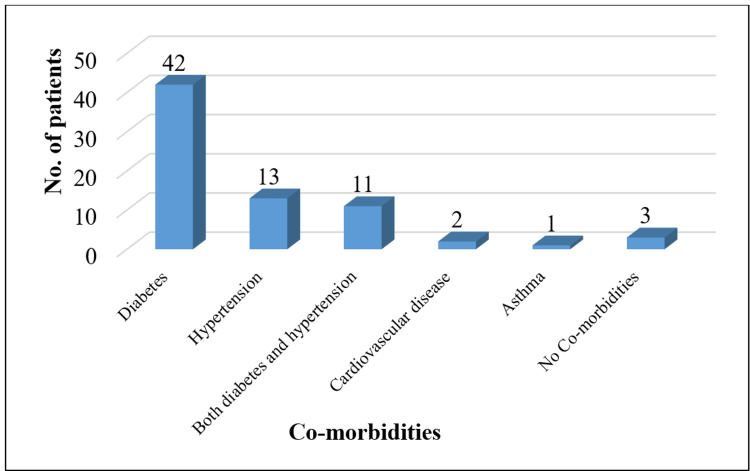
Comorbidities in post-COVID‑19 mucormycosis patients These categorical variables were summarized as frequencies.

The most common comorbidity was diabetes mellitus found in 42 (58.33%) patients, followed by hypertension (n=13; 18.05%) and both diabetes with hypertension (n=11; 15.27%). Only two patients (2.77%) had a history of cardiovascular disease. Asthma was noted in one patient. Three patients (4.16%) did not have any reported comorbidities (see Figure 2).

Surgical debridement was necessary for 45 patients, accounting for 62.5%. All 72 patients (100%) received Amphotericin B, an antifungal medication essential for treating mucormycosis. Out of 72 patients, 40 (55.6%) survived, and 32 (44.4%) patients died (see Table 2).

**Table 2:** Clinical outcomes These categorical variables were summarized as frequencies and percentages

Clinical outcomes	Frequency	Percentage
Required surgical debridement	45	62.5
Received Amphotericin B	72	100.0
Survived	40	55.6
Deceased	32	44.4

The mean age of survivors was 52.31±11.25 years, while non-survivors had a mean age of 58.33±13.22 years, which was statistically significant with a p-value of 0.041 (one-way ANOVA), indicating that older age was associated with higher mortality (see Table 3). Among survivors, 60% of patients (24 patients) had diabetes mellitus, compared to 75% of non-survivors (24 patients). Although a higher percentage of non-survivors had diabetes, the difference was not statistically significant, with a p-value of 0.15 (chi-square test). Corticosteroid use was reported in 27 survivors (67.5%) and 18 non-survivors (56.25%), which was not statistically significant (p=0.327; chi-square test). Early surgical intervention was performed in 30 survivors (75.0%) compared to 15 non-survivors (46.9%). This difference was statistically significant with a p-value of 0.01 (chi-square test), suggesting that early surgical intervention was associated with improved survival. Thus, data indicates that older age and lack of early surgical intervention were significant factors associated with higher mortality in patients with mucormycosis in COVID-19.

**Table 3:** Factors influencing mortality * One-way ANOVA for mean age where p<0.001 was considered significant and the chi-square test for other categorical variables where p<0.05 was considered significant.

Factors	Survivors (n=40)	Non-survivors (n=32)	Test statistic value	p-value
Mean age (years), mean±SD	52.31±11.25	58.33±13.22	4.356	0.041*
Diabetes mellitus, n (%)	24 (60.0%)	24 (75.0%)	1.8	0.179
Use of corticosteroid, n (%)	27 (67.5%)	18 (56.25%)	0.96	0.327
Early surgical intervention, n (%)	30 (75.0%)	15 (46.9%)	6.0	0.014*

## Discussion

The present study analyzed the 72 patients diagnosed with mucormycosis following COVID-19 infection, with a particular focus on demographic characteristics, clinical profile, comorbidities, and outcomes. The majority of patients were in the age group of 51 to 60 years (n=34; 43.05%), with a mean age of 55.42±12.31 years. This finding is consistent with a previous study conducted by Patel et al. (2021), where they found that the mean age of mucormycosis patients was 54.5 years [11], indicating that middle-aged to older adults are predominantly affected. The male predominance observed in the present study (n=44; 61.11%) is also in line with findings from another research done by Singh et al. (2021), which reported a male-to-female ratio of approximately 3:1 [12].

The clinical features of mucormycosis include facial pain, nasal congestion, orbital swelling, and necrotic lesions. In severe cases, the infection can extend to the brain, leading to neurological complications and death. Similarly, in the present study, facial pain (n=61; 84.72%) and headache (n=58; 80.55%) were the most common clinical features. These symptoms are consistent with the clinical presentation of mucormycosis reported in Balushi et al. (2022) study [13].Most patients exhibited more than one symptom, which is indicative of the multifaceted nature of mucormycosis and its interaction with COVID-19. The mean duration of symptoms in the current study was 14.31±5.4 days. This is comparable to the findings of Singh et al. (2021), who reported a mean symptom duration of approximately 13 days in mucormycosis patients [12]. The prolonged duration of symptoms underscores the aggressive nature of mucormycosis and the need for timely medical intervention.

The corticosteroids were used in a significant number of patients (n=50; 69%) as part of their COVID-19 treatment regimen. The widespread use of corticosteroids is a well-documented factor in the increased incidence of mucormycosis among COVID-19 patients. Corticosteroids, while effective in reducing inflammation, can impair the immune response and increase blood glucose levels, creating an environment conducive to fungal infections. Similarly, Balushi et al. (2022) found that corticosteroid use was prevalent in 50% of their study cohort, reflecting the role of these drugs in predisposing patients to secondary fungal infections [13]. T​​he findings from this study corroborate the established link between corticosteroid use and the risk of developing mucormycosis in COVID-19 patients.

The high prevalence of diabetes mellitus (n=42; 58.33%) among the patients in the current study underscores its role as a significant risk factor for mucormycosis, particularly in the context of COVID-19. Previous research has similarly highlighted the strong association between diabetes and mucormycosis [14,15]. Chakravarty et al. (2022) reported that 95.7% of mucormycosis patients had diabetes, reinforcing the critical need for stringent glycemic control in COVID-19 patients to mitigate the risk of mucormycosis [16]. Hypertension was the second most common comorbidity (n=13; 18.05%), followed by a combination of diabetes and hypertension (n=11; 15.28%). These findings are consistent with those of Song et al. (2020), who noted a high prevalence of hypertension among mucormycosis patients [17]. The relatively lower frequency of other comorbidities, such as cardiovascular disease (n=2; 2.77%) and asthma (n=1; 1.38%) in this study is comparable to other studies [14,15], indicating that while these conditions are present, they are not as prevalent as diabetes and hypertension.

Mucormycosis is an angioinvasive fungal infection characterized by rapid progression and high mortality rates [18]. The present study revealed a mortality rate of 44.4%, which is within the range reported by other studies. A study done by Balushi et al. found a mortality rate of 60% among mucormycosis patients [13]. The high mortality rate underscores the severe nature of mucormycosis, particularly in immunocompromised individuals such as those recovering from COVID-19.

Early diagnosis and prompt surgical intervention were identified as crucial factors in improving patient outcomes. In the current study, 62.5% of patients (30 patients) required surgical debridement, which aligns with the findings of Sen et al. (2021), who emphasized the importance of combined surgical and antifungal therapy in managing mucormycosis [19]. Also, our findings are comparable with the study conducted by Patel et al. (2021) [11] and Singh et al. (2021) [12]. The similarities in findings reinforce the generalizability of the risk factors and outcomes associated with mucormycosis, while the regional focus highlights the importance of localized healthcare strategies to manage this dual infection effectively.

As a retrospective study, there is a potential for incomplete or missing data. Patient records may not have all the necessary information, leading to gaps in the data collection process. The study was conducted at a single tertiary care hospital, which may limit the generalizability of the findings. There may be other confounding variables that were not accounted for in the study, which could influence the outcomes. For example, the severity of COVID-19, pre-existing health conditions, and variations in treatment protocols could all affect the results.

## Conclusions

The present study indicates a high mortality rate among COVID-19 patients with mucormycosis, particularly in older patients and those who did not receive early surgical intervention. Diabetes mellitus and corticosteroid use were prevalent among the patients but were not statistically significant factors for mortality. The universal use of amphotericin B highlights its importance in managing mucormycosis, but the timing and combination with surgical debridement were crucial for better outcomes.

This retrospective study reinforces the critical role of early diagnosis, immediate systemic antifungal therapy, stringent management of comorbidities, and prompt surgical intervention in improving outcomes for mucormycosis in COVID-19 patients. Early surgical intervention significantly improves survival rates, emphasizing the importance of prompt and comprehensive management strategies. Further research is needed to establish standardized treatment protocols and preventive measures for better management of mucormycosis.
